# Tubular reabsorption and local production of urine hepcidin-25

**DOI:** 10.1186/1471-2369-14-70

**Published:** 2013-03-25

**Authors:** Hilde PE Peters, Coby MM Laarakkers, Peter Pickkers, Rosalinde Masereeuw, Otto C Boerman, Annemarie Eek, Elisabeth AM Cornelissen, Dorine W Swinkels, Jack FM Wetzels

**Affiliations:** 1 Nephrology, Radboud University Nijmegen Medical Centre, Nijmegen, The Netherlands; 2 Laboratory Medicine, Laboratory of Genetic, Endocrine and Metabolic Diseases, Radboud University Nijmegen Medical Centre, Nijmegen, The Netherlands; 3 Intensive Care Medicine, Radboud University Nijmegen Medical Centre, Nijmegen, The Netherlands; 4 Pharmacology and Toxicology, Radboud University Nijmegen Medical Centre, Nijmegen, The Netherlands; 5 Nuclear Medicine, Radboud University Nijmegen Medical Centre, Nijmegen, The Netherlands; 6 Pediatric Nephrology, Radboud University Nijmegen Medical Centre, Nijmegen, The Netherlands

**Keywords:** AKI, β_2_-microglobulin, Hepcidin, Megalin, Kidney tubules

## Abstract

**Background:**

Hepcidin is a central regulator of iron metabolism. Serum hepcidin levels are increased in patients with renal insufficiency, which may contribute to anemia. Urine hepcidin was found to be increased in some patients after cardiac surgery, and these patients were less likely to develop acute kidney injury. It has been suggested that urine hepcidin may protect by attenuating heme-mediated injury, but processes involved in urine hepcidin excretion are unknown.

**Methods:**

To assess the role of tubular reabsorption we compared fractional excretion (FE) of hepcidin-25 with FE of β2-microglobulin (β_2_m) in 30 patients with various degrees of tubular impairment due to chronic renal disease. To prove that hepcidin is reabsorbed by the tubules in a megalin-dependent manner, we measured urine hepcidin-1 in wild-type and kidney specific megalin-deficient mice. Lastly, we evaluated FE of hepcidin-25 and β_2_m in 19 patients who underwent cardiopulmonary bypass surgery. Hepcidin was measured by a mass spectrometry assay (MS), whereas β_2_m was measured by ELISA.

**Results:**

In patients with chronic renal disease, FE of hepcidin-25 was strongly correlated with FE of β_2_m (r = 0.93, P <0.01). In megalin-deficient mice, urine hepcidin-1 was 7-fold increased compared to wild-type mice (p < 0.01) indicating that proximal tubular reabsorption occurs in a megalin- dependent manner. Following cardiac surgery, FE of hepcidin-25 increased despite a decline in FE of β_2_m, potentially indicating local production at 12–24 hours.

**Conclusions:**

Hepcidin-25 is reabsorbed by the renal tubules and increased urine hepcidin-25 levels may reflect a reduction in tubular uptake. Uncoupling of FE of hepcidin-25 and β_2_m in cardiac surgery patients suggests local production.

## Background

Hepcidin, a peptide predominantly produced by hepatocytes, is a major player in iron metabolism [1,2]. Hepcidin decreases duodenal iron absorption and causes iron sequestration in the reticulo-endothelial system [3,4]. Hepcidin expression is induced by iron storage and inflammation [5,6] and suppressed by hypoxia and anemia [5]. Serum hepcidin levels are increased in patients with renal insufficiency [7,8], and this may contribute to anemia and resistance to erythropoietin stimulating agents.

Recent studies have pointed to the relevance of urine hepcidin. In patients with lupus nephritis, changes in urine hepcidin-20 and −25 predicted renal flares [9]. Even more striking were the findings of Ho *et al.*, who showed that patients with *increased* urine hepcidin levels were at *lower* risk to develop acute kidney injury (AKI) after cardiac surgery [10,11]. These results have recently been confirmed in a larger study that included 100 patients who had undergone cardiopulmonary bypass surgery (CABG) [12]. It was suggested that urine hepcidin may protect against AKI by attenuating heme-mediated injury.

In order to meaningfully interpret urine hepcidin as a biomarker, knowledge of renal handling is essential. Thus far, it is unclear which processes – filtration, reabsorption, local production and/or degradation- govern urine hepcidin excretion. The objective of this study was to study the role of tubular reabsorption in kidney hepcidin handling. Our data provide evidence for both tubular reabsorption and local production of hepcidin in the kidney.

## Methods

### Human studies

Blood and urine samples of healthy controls were collected randomly throughout the day as described before [13]. In order to assess the role of tubular reabsorption we compared fractional excretion (FE) of hepcidin-25 with FE of β_2_-microglobulin (β_2_m) in patients with glomerular or tubular diseases and various degrees of impairment of tubular reabsorption. β_2_m is a low molecular weight protein and an established marker of proximal tubular function. Patients were enrolled from February 2009 to October 2010 at the Department of Nephrology and Pediatric Nephrology, Radboud University Nijmegen Medical Center. Patients with biopsy proven glomerular disease (without an interstitial infiltrate) and patients with defined tubular diseases (cystinosis, Dent’s disease) were enrolled. Patients with an interstitial infiltrate as concluded from renal biopsy investigations were excluded, since monocytes may produce hepcidin [14].

Hepcidin-25 was also measured in patients after cardiopulmonary bypass surgery. Patients were enrolled consecutively from March 2008 to April 2008 at the Department of Intensive Care Medicine, Radboud University Nijmegen Medical Centre, and all patients undergoing CABG were included. Serum and urine samples were obtained simultaneously either 1–2 hours after surgery (at a time point the patient was admitted and stable at the ICU) and 12–24 hours after the end of surgery (morning urine collected the day after the procedure).

The study has been carried out in the Netherlands in accordance with the applicable rules concerning the review of research ethics committees and informed consent. We obtained consent from healthy volunteers. The local ethics committee waived the need to get consent from patients as they were having blood and urine taken as part of standard care.

### Animal experiments

In order to investigate whether hepcidin is reabsorbed in the proximal tubules in a megalin-dependent manner, we measured urine hepcidin-1 in wild type and kidney specific megalin-deficient C57Bl/6 mice [15]. Megalin lox/lox; apoECre mice on a C57BL/6 background were kindly provided by Thomas E. Willnow. The creation of this kidney specific megalin-deficient mouse strain was described in detail previously [15]. Animals were bred locally and animals expressing the apoECre gene were identified by means of polymerase chain reaction (PCR) analysis. Animals that did not express the apoECre gene (megalinlox/lox mice) were used as wild type controls. Approximately 12 weeks old female mice were used. Their diet contained 179 mg/kg iron. To collect urine samples, mice were individually housed in metabolic cages (Techniplast®). Mice were allowed to adapt to these cages during 2 periods of 30 min, after which 24-hour urine samples were collected. Prior to 24 h housing in metabolic cages, 2×0.5 ml salt solution was administered subcutaneously to prevent dehydration. To prevent hypothermia, room temperature was raised to 24°C with a relative humidity of 53-68%. Food and water were available ad libitum. Mice were sacrificed after blood sampling at the end of the 24-hour urine collection. Urinary protein profile of wild-type and megalin deficient mice was determined by gel electrophoresis.

Experiments were approved by the Animal Ethical Commission of the Radboud University Nijmegen Medical Centre and performed in accordance with national guidelines for the care and handling of animals.

### Laboratory measurements

Urine and serum samples were processed, aliquoted, and stored in polypropylene tubes at −80°C immediately after collection. Routine laboratory parameters and hepcidin levels were measured within 8 hours and 6 months of collection, respectively.

Urinary and serum creatinine were measured with an enzymatic method. Urine β_2_m was measured by ELISA. Urine β_2_m was only measured in urine with a urinary pH >6.0, since degradation of β_2_m may occur below this pH. Human hepcidin-25 was measured by our previously described weak cation exchange time-of-flight mass spectrometry assay [16] (TOF MS). Hepcidin-1 in urine from mice was measured by surface-enhanced laser desorption ionization (SELDI) TOF MS [17].

#### Calculations and statistics

Depending on its distribution, data were expressed as median (interquartile range) or mean ± standard deviation (SD). Fractional excretion of substance Y was defined as: (Serum Creatinine × Urine Y) / (Serum Y × Urine Creatinine) × 100%. In healthy controls, serum and urine hepcidin-25 were expressed as the mean of four samples collected at different times during the day. In mice urinary concentrations were normalized for creatinine to correct for differences in urine dilution.

Glomerular filtration rate (GFR) was estimated by the abbreviated Modification of Diet in Renal Disease equation in adults [18]. In children GFR was estimated using the revised Schwartz formula [19].

Statistical analysis was performed using SPSS 16.0 (SPSS Inc, Chicago, IL). Correlations were assessed by linear regression, using Spearman’s rho. Mann Whitney test was used for comparison of FE of hepcidin-25 in healthy controls and patients with renal disease and for comparison of urinary concentrations in wild type and megalin-deficient mice. Statistical significance was denoted by two sided P values of <0.05.

## Results

**Figure 1:**
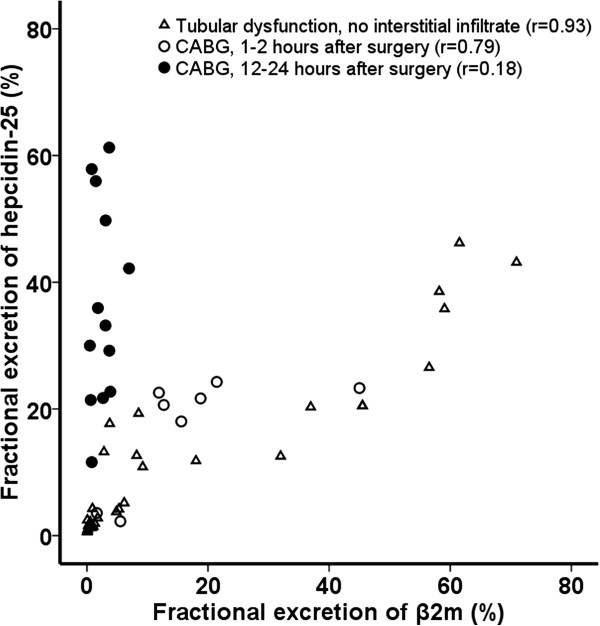
**Correlation between fractional excretion (FE) of hepcidin-25 and β2-microglobulin (β2m) in patients with renal disease without a tubulointerstitial infiltrate (n = 30, triangles), and in patients at 1–2 hours (n = 8, white circles) and 12–24 hours (n = 13, black circles) after cardiopulmonary bypass grafting (CABG).** FE of hepcidin correlates with FE of β2m, indicating tubular uptake of hepcidin. However, FE of hepcidin increased relatively more in patients 12–24 hours after CABG, suggesting local production in the kidney.

In 24 healthy controls fractional excretion (FE) of hepcidin-25 was 1.9 (IQR 1.0-3.2)% (Table 1). We evaluated 30 patients with glomerular or tubular diseases and various degrees of impairment in tubular reabsorption. Median serum creatinine was 107 (IQR 83–147) μmol/l and proteinuria 4.5 (IQR 1.7-9.4) g/d (Table 1). Renal disease consisted of idiopathic membranous nephropathy (n = 13), focal segmental glomerulosclerosis (n = 3), infantile nephropathic cystinosis (n = 7), or other causes (n = 7). There was an increased FE of hepcidin-25 in patients with renal disease compared to controls (8.0 versus 1.9%, p < 0.001). We found a strong correlation between FE of hepcidin-25 and FE of β_2_m, Spearman’s rho = 0.93, p < 0.01, Figure 1). Since β_2_m is a marker of proximal tubular reabsorption, this data strongly suggest that hepcidin excretion is at least partially governed by this process.

**Table 1:** Clinical and demographic characteristics

**Variable**	**Controls (n = 24)**	**Renal disease (n = 30)**	**CABG 1–2 hours (n = 8)**	**CABG 12–24 hours (n = 13)**
**Male (%)**	46	80	88	85
**Age (years)**	39 ± 12	43 ± 24	65 ± 8	66 ± 10
**Serum albumin (g/l)**	n.a.	34 (24–35)	n.a.	n.a.
**Serum creatinine (μmol/l)**	75 (68–83)	107 (23–147)	93 (74–116)	85 (51–102)
**eGFR (ml/min/1.73 m**^**2**^**)**	84 (77–108)	57 (34–65)	73 (58–95)	85 (17–128)
**Proteinuria (g/d)**	n.a.	4.5 (1.7-9.4)	n.a.	n.a.
**Serum β**_**2**_**m (mg/l)**	n.a.	2.9 (2.2-4.6)	2.1 (1.6-2.9)	1.8 (1.3-2.5)
**Serum hepcidin-25 (nmol/l)**	4.4 (3.2-5.8)	3.1 (1.1-5.7)	4.7 (1.5-7.1)	14.1 (11.1-17.8)
**Urine β**_**2**_**m (nmol/mmol creatinine)**	n.a.	142 (13–863)	312 (1528–5239)	66 (21–86)
**Urine hepcidin-25 (nmol/mmol creatinine)**	0.9 (0.4-1.7)	1.4 (0.3-7.1)	10.4 (0.8-18.7)	52.2 (34.0-112.1)
**FE of β**_**2**_**m (%)**	n.a.	5.1 (0.7-39.1)	14.1 (7.1-20.7)	2.7 (0.8-3.7)
**FE of hepcidin-25 (%)**	1.9 (1.0-3.2)	8.0 (1.9-20.3)	21.1 (7.2-23.1)	33.1 (22.2-52.9)
**Duration of CPB (min)**	-	-	101 (63–147)^#^	106 (92–125)^#^

Median (interquartile range), mean ± SD, eGFR = estimated glomerular filtration rate, n.a. = not available, FE = fractional excretion, CABG = coronary artery bypass grafting, CPB = cardiopulmonary bypass, ^#^ one patient underwent off-pump CABG.

**Figure 2:**
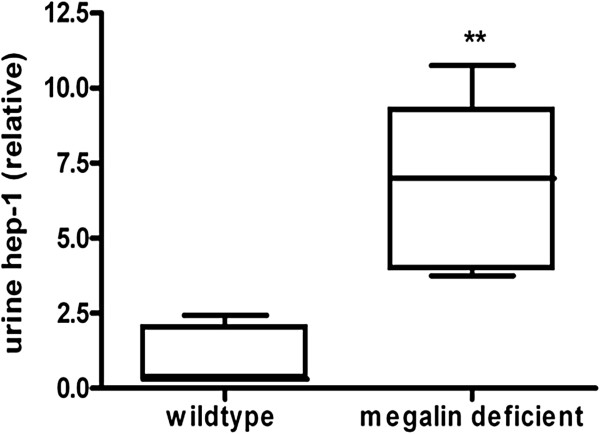
**Hepcidin-1 in urine of C57Bl/6 Wild type mice (n = 5) and mice with kidney-specific megalin deficiency (n = 5).** Urine was normalized for creatinine levels. Data are depicted as lower quartile, median and upper quartile (boxes), and minimum and maximum ranges (whiskers). **,P < 0.01;megalin deficient vs. wild type mice (by Mann Whitney test).

Megalin is a multiligand endocytic receptor localized in the proximal renal tubules and plays an important role in the tubular reabsorption of various filtered proteins, amongst which β_2_m. To prove that the bioactive mouse hepcidin-1 is reabsorbed in the proximal tubules in a megalin-dependent manner, we measured urine hepcidin in wild type and kidney specific megalin-deficient C57Bl/6 mice. As expected, megalin deficient mice did excrete low molecular weight proteins (Additional file 1 Urinary protein profile of wild-type and megalin deficient mice, web appendix). Urine hepcidin-1 was 7-fold increased in megalin-deficient mice (n = 5) compared to wild type mice (n = 5, Figure 2; p < 0.01). Of note, glomerular filtration was not affected in megalin-deficient mice compared to wild type mice (creatinine clearance 205 ± 146 versus 249 ± 24 μl/min, NS).

We next evaluated urine hepcidin-25 in patients who underwent cardiopulmonary bypass surgery (Table 1). 24 patients were initially enrolled, but 5 patients were excluded because of a urinary pH <6 at both time intervals, thus precluding reliable measurement of β_2_m. All remaining 19 patients were operated on pump, except for one patient who had pre-existing impairment of renal function. Two patients developed acute kidney injury after cardiac surgery, with AKI defined as a baseline-to-peak decrease in eGFR by 50% or more during the first five days post-operatively. pH was >6 in approximately 50% of measurements performed in these 19 patients , allowing serum and urine β_2_m and hepcidin quantification in eight patients, 1–2 hours after surgery, and in 13 patients 12–24 hours after surgery. In only 2 patients measurements of β_2_m and hepcidin could be performed at both time intervals.

**Figure 3:**
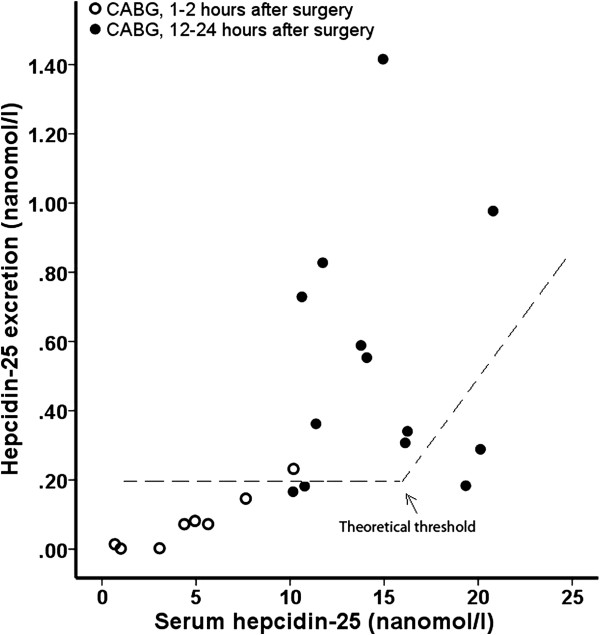
**Serum hepcidin-25 versus estimated urinary excretion of hepcidin-25 in patients 1–2 and 12–24 hours post surgery.** There is no correlation indicating a threshold of serum hepcidin-25 above which urinary excretion steeply increases. Spearman’s rho is 0.91 for 8 patients at day 1 and 0.16 for patients at day 2.

Immediately after surgery, FE of hepcidin-25 and β_2_m were 21 and 14%, respectively, and correlated strongly (Spearman’s rho = 0.79, p = 0.02). The ratio between both parameters was similar to that in patients with renal disease, and thus compatible with impairment of tubular reabsorption. At 12–24 hours after surgery FE β_2_m decreased (3%), indicating recovery from tubular injury. However, FE of hepcidin-25 increased further (33%). As a result, FE of hepcidin-25 did no longer correlate with FE of β_2_m (Spearman’s rho = 0.18, p = 0.55, Figure 1). When plotting serum hepcidin versus urinary excretion of hepcidin (or GFR*serum hepcidin vs urinary excretion of hepcidin) there was no evidence of a tubular threshold (Figure 3).

## Discussion

Our results indicate that hepcidin-25 is reabsorbed by the renal proximal tubules via megalin-mediated endocytosis. In addition, our data suggest that the increase of urine hepicidin-25 after cardiac surgery reflects local production of hepcidin in the kidney.

Urine hepcidin has recently gained interest as a renal biomarker. However, not much is known about the renal handling of hepcidin. Because of its small size (2.8 kDa), free, unbound hepcidin is likely to be filtered by the glomeruli. After filtration, hepcidin is almost completely reabsorbed by the renal tubules. This conclusion is based on data from a small number (n = 9) of healthy controls and patients with thalassemia and hemochromatosis, where FE of hepcidin was estimated to be less than 3% [20,21].

In the present study, FE of hepcidin-25 in healthy controls was approximately 2%, thus confirming earlier reports and compatible with the suggested extensive tubular uptake [20,21]. In patients with kidney diseases, FE of hepcidin-25 was increased and correlated strongly with FE of β_2_m (r = 0.93, p < 0.01), a low molecular weight protein that is normally freely filtered by the glomerulus and almost completely reabsorbed in the proximal tubule through the megalin receptor. By using a mouse model, we confirmed the existence of megalin-dependent tubular reabsorption of hepcidin-25. Unlike humans, mice contain two related hepcidin genes, hepcidin-1 and 2, of which hepcidin-1 is almost exclusively produced in the liver. This peptide is important for iron homeostasis, and is considered the mouse equivalent of human hepcidin-25 [3,22,23]. Urine hepcidin-1 was significantly higher in megalin-deficient mice (p < 0.01, Figure 2).

We observed that shortly after cardiac surgery, FE of both hepcidin-25 and β_2_m were increased and correlated strongly, reflecting decreased tubular reabsorption. However, within 24 hours after surgery we observed a further increase of FE of hepcidin-25, whereas at this time point tubular injury became less severe as shown by the reduction in FE of β_2_m. These findings can be explained in two ways; either by the occurrence of saturation of tubular reabsorption due to increased tubular delivery of hepcidin-25 thus exceeding reabsorption capacity or by production of hepcidin-25 locally in the kidney. Since we did not observe a threshold (Figure 3), local production is the most likely explanation.

Data on tubular reabsorption or local production are scarce. Kulaksiz *et al.* observed strong hepcidin expression in the thick ascending limb of the cortex and in the connecting tubules, but not in the proximal tubules [24]. At the cellular level, hepcidin was localized to the apical cell pole of the renal epithelial cells, which is suggestive of luminally directed release of hepcidin in the urine. Hepcidin-25 may thus be produced by the distal kidney tubules [24] due to unknown stimuli or by inflammatory cells such as monocytes [14].

The origin and regulation of locally produced hepcidin-25 in patients after cardiac surgery merits further studies in view of the recent evidence that urine hepcidin may protect against the development of AKI. In a nested cohort study Ho *et al.* compared urine of 22 cardiac surgery patients with AKI (defined as ≥50% rise in serum creatinine during the first four postoperative days) with urine from 22 randomly selected cardiac surgery patients without AKI [10]. They observed that hepcidin-25 was increased on the first post-operative day in patients *not* developing AKI. The observations of Ho *et al.* were corroborated by an independent observational study measuring hepcidin through ELISA in 100 cardiac surgery patients [12]: urine hepcidin was 3–7 times higher 6 and 24 hours after surgery in AKI-free patients (n = 91) compared to 9 patients who developed AKI (defined as ≥50% rise in serum creatinine or urine output <0.3 ml/kg/hr during the first seven postoperative days). Additionally, FE of hepcidin increased from 8 to 40% at 24 h post surgery in 93 patients exposed to cardiac surgery, and was higher in patients who did not develop AKI (AKI (n = 25) 27% vs AKI-free (n = 68) 37%, p = 0.049) [25].

As suggested by others [26], local production of hepcidin may serve to prevent oxidative damage induced by free iron and thereby protect against AKI. Some studies have reported that hepcidin binds divalent metals, amongst which Fe2^+^[27,28].

This study is the first to document local production of hepcidin in patients after CABG. It has several limitations. First, we included a limited number of patients. Secondly, due to a pH <6.0 β2m could not reliably be measured in all samples. Although alfa-1 microglobulin is a more stable marker of proximal tubular reabsorption and can reliably be measured in acidic urine, it is protein-bound and therefore it is impossible to calculate the fractional excretion. Third, this study is a pilot study, and our findings were not corroborated by histopathological data showing extensive proximal tubular uptake in apical endocytic vesicles, nor by data on hepcidin expression or mRNA content in the kidney or macrophages. More extensive studies are necessary to evaluate the exact timing and location of hepcidin production, and to identify possible factors influencing this process. Since increased urinary levels of hepcidin are associated with a decreased risk for post-surgical AKI, increasing local production may serve as a strategy to reduce the development of AKI.

## Conclusion

In conclusion, our mouse study indicates that proximal tubular reabsorption of urine hepcidin-1 occurs in a megalin-dependent manner. In CKD patients FE of hepcidin-25 correlated strongly with FE of β_2_m, suggesting that also in human urinary excretion of hepcidin-25 is governed by tubular reabsorption of hepcidin. Uncoupling of FE of hepcidin-25 and β_2_m in cardiac surgery patients indicates local production of hepcidin-25. This local production of hepcidin-25 may be important in attenuating post-surgical AKI and merits further investigation.

## Competing interests

We have nothing to disclose. DWS is a co-founder and Medical Director of the ‘Hepcidinanalysis.com’ initiative, which aims to serve the scientific and medical community with high-quality human and animal hepcidin measurements (http://www.hepcidinanalysis.com).

## Authors’ contributions

HP participated in the design of the study, performed the statistical analysis and wrote the manuscript, CL carried out mass spectrometry measurements of hepcidin and made substantial contributions to data interpretation and analysis,PP participated in interpretation of data, RM and AE carried out mice studies and participated in the interpretation of data, EC contributed to collecting and interpretation of the data, DS participated in the design of the study and measurements of hepcidin and helped to draft the manuscript, JW participated in the design of the study, interpretation of data and helped to draft the manuscript. All authors read and approved the final manuscript.

## Short summary

We demonstrate that urinary excretion of hepcidin is dependent on tubular reabsorption. However, we provide evidence that increased levels of urine hepcidin after cardiac surgery are explained by local production. Local hepcidin production may be important in attenuating post-surgical acute kidney injury.

## Pre-publication history

The pre-publication history for this paper can be accessed here:

http://www.biomedcentral.com/1471-2369/14/70/prepub

## Supplementary Material

Additional file 1 Urinary protein profile of wild-type and megalin deficient mice. Click here for file
