# Supplementary figures and images for: Tubular reabsorption and local production of urine hepcidin-25
Source: BMC Nephrol. 2013 Mar 25;14:70. doi: 10.1186/1471-2369-14-70 (PMC3623618; doi:10.1186/1471-2369-14-70)

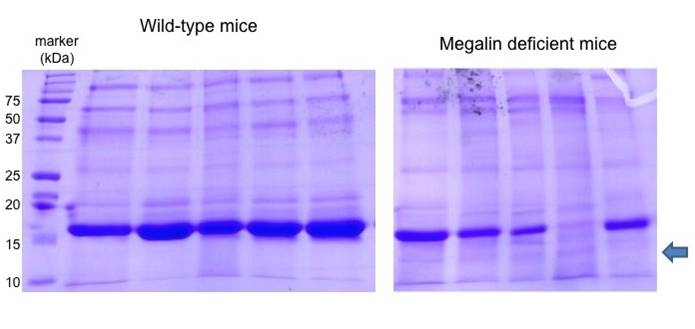

Supplement: Additional file 1 — Urinary protein profile of wild-type and megalin deficient mice. [file 1471-2369-14-70-S1.jpeg]
